# Fever duration, peak temperature and associated symptoms in Chinese adults with SARS-CoV-2 infection: a cross-sectional study

**DOI:** 10.3389/fmed.2025.1665460

**Published:** 2025-11-06

**Authors:** Yang Ye, Xiyan Xin, Xinying Du, Yunjing Bai, Hongbo Shen

**Affiliations:** 1 Department of Traditional Chinese Medicine, Peking University Third Hospital, Beijing, China; 2 Department of Rheumatism and Immunology, Seventh Medical Center of PLA General Hospital, Beijing, China

**Keywords:** SARS-CoV-2, fever, gender, age, cross-sectional survey

## Abstract

**Introduction:**

Although the COVID-19 pandemic has passed, we still lack a comprehensive understanding of its fever-related characteristics. This study aims to elucidate the unique fever characteristics exhibited by Chinese SARS-CoV-2 patients, providing insights into the fever patterns of COVID-19 and enhancing our understanding of fever caused by viral infections.

**Methods:**

A descriptive study was conducted using an online questionnaire administered through a web-based survey platform from December 19, 2022, to January 5, 2023. Data pertaining to the demographic features and fever dynamics of SARS-CoV-2-infected individuals were collected and analyzed.

**Results:**

The study cohort consisted of 555 adult individuals presenting with fever, comprising 157 males (28.3%) and 398 females (71.7%), with a mean age of 39.8 ± 12.7 years. The average duration of fever was 2.5 ± 1.6 days, with a peak temperature recorded at 38.7 ± 0.7 °C. Notably, a negative correlation was discerned between peak fever temperature and age, while in female participants, fever duration displayed a positive correlation with age. Patients with underlying comorbidities, particularly those with lupus erythematosus, exhibited lower peak temperatures. Predominant symptoms reported during febrile episodes encompassed headache, cough, general malaise, and myalgia, while post-fever symptoms predominantly comprised cough, sputum production, nasal obstruction, and sore throat.

**Conclusion:**

Age may be an important factor influencing both the duration of fever and peak body temperature in SARS-CoV-2 infected individuals, while gender-specific variations appear to be limited. COVID-19 patients demonstrated noticeable heterogeneity in symptom manifestations both during the febrile phase and after fever resolution.

## 1 Introduction

The coronavirus disease 2019 (COVID-19) pandemic, caused by severe acute respiratory syndrome coronavirus 2 (SARS-CoV-2), was a significant global health challenge (1). By December 2022, the World Health Organization reported that the virus had infected over 640 million individuals worldwide (2). The public health crisis stemming from COVID-19 has led to widespread societal anxiety, strained healthcare resources, and subsequent social disruption (3). Clinically, COVID-19 is characterized by a range of influenza-like symptoms, including fever, cough, pharyngitis, rhinorrhea, and headache, with fever being the most commonly reported symptom (4, 5). However, a comprehensive understanding of the unique fever patterns exhibited by COVID-19 patients remains elusive.

An elevated body temperature above 37.2 °C (99°F) traditionally indicates the presence of fever, a pathological condition commonly triggered by bacterial or viral infections (6). Accompanied by symptoms such as flushed cheeks, increased warmth upon palpation, and general malaise, fever represents a fundamental immunological response aimed at eliminating infectious agents (7). Fever is a prominent clinical feature observed in patients with SARS-CoV-2, affecting ~51% of cases (8). Data from a retrospective cohort analysis reveal that the mean age of individuals with SARS-CoV-2 infection is 45 years, with 25.8% experiencing fever (9). Furthermore, research suggests a lower prevalence of fever among younger cohorts diagnosed with mild to moderate forms of COVID-19 (10). Studies also indicate that elderly COVID-19 patients tend to have lower baseline temperatures and achieve lower peak temperatures during their illness trajectory (11). Investigative findings suggest that non-febrile COVID-19 patients are generally older and present with a higher incidence of concurrent morbidities (12). Additionally, a study among hospitalized patients in Ghana has identified a correlation between fever incidence and disease severity escalation in COVID-19 cases (13).

While there have been studies focusing on the thermal profiles of patients with COVID-19, these studies have produced limited findings that do not fully explain the specific fever patterns of this patient population. Particularly, there is a noticeable lack of research on Chinese cohorts. In response, our study initiates a web-based cross-sectional analysis designed to investigate the fever characteristics of individuals suffering from SARS-CoV-2 in China. The purpose of this study is to deepen our understanding of fever manifestations in Chinese COVID-19 patients, offering new insights for healthcare professionals, epidemiologists, and readers to comprehend the fever patterns of COVID-19.

## 2 Methods

### 2.1 Design and participants

This investigation was carried out in China from December 19, 2022, to January 5, 2023. The data collection process was diligently executed utilizing a reliable online survey platform tailored for questionnaire dissemination and collection. The scope of the survey was precisely delimited to incorporate respondents who had been clinically diagnosed with COVID-19 and exhibited fever as a symptom. The inclusionary parameters were rigorously defined as follows: (1) confirmed SARS-CoV-2 infection evidenced by either nucleic acid amplification tests or antigen testing modalities, (2) a recorded peak body temperature of ≥ 37.2 °C (6) concurrent with the period of infection, and (3) an age demographic bracket of 18–70 years for participants. Conversely, the criteria for exclusion were stringently set to ensure data quality, rejecting (1) overtly erroneous responses (e.g., age > 150 years or body temperature >45 °C, which are biologically implausible), as well as (2) submissions with missing or unclear key information. In China, body temperature is typically assessed using either an underarm thermometer or an infrared thermometer. The research did not gather any personally identifiable information that could potentially compromise privacy. This study adheres to the Declaration of Helsinki and has been approved by the Medical Science Research Ethics Committee of Peking University Third Hospital. Tacit informed consent was obtained from all study participants.

### 2.2 Questionnaires

The survey was disseminated to respondents via the WeChat Questionnaire Star platform (14), utilizing a convenience sampling approach. It comprised a comprehensive questionnaire encompassing nine items addressing variables such as gender, age, duration of fever, onset time, peak body temperature, presence of comorbidities, initial symptoms, and post-fever symptoms. The questionnaire featured a combination of multiple-choice and open-ended questions to capture a spectrum of responses. Respondents were required to designate complications (e.g., coronary heart disease, hypertension, diabetes, rheumatoid arthritis, Sjogren's syndrome), symptoms at onset (e.g., fever, cough, body pain, sore throat, fatigue), and primary symptoms following fever resolution (e.g., cough, phlegm, dry throat, loss of smell, loss of taste). Parameters such as age, duration of fever, onset time, and maximum body temperature were elicited through open-ended responses. Data on submission time, duration of completion, and participants' Internet Protocol (IP) addresses were automatically captured. The questionnaire's content underwent rigorous scrutiny and peer discussion within the research team to ensure its methodological rigor. Prior to the formal launch of the survey, internal pilot testing was conducted to enhance usability and precision, facilitating a more streamlined and accurate data gathering process.

### 2.3 Statistical analysis

Data analysis was conducted utilizing GraphPad Prism 9.5.1 software. Descriptive statistics were employed to represent normally distributed variables in the format of Mean ± SD. Group comparisons were carried out using unpaired sample *t*-tests for binary group comparisons, while one-way or two-way analysis of variance (ANOVA) was utilized for comparisons among multiple groups. Chi-square test was used for count data. Standard two-way ANOVA model was utilized to assess potential interaction effects between age and sex. All statistical tests were two-tailed, with a significance threshold set at *p* < 0.05 indicating statistical significance.

## 3 Results

### 3.1 Participant characteristics

**Figure 1:**
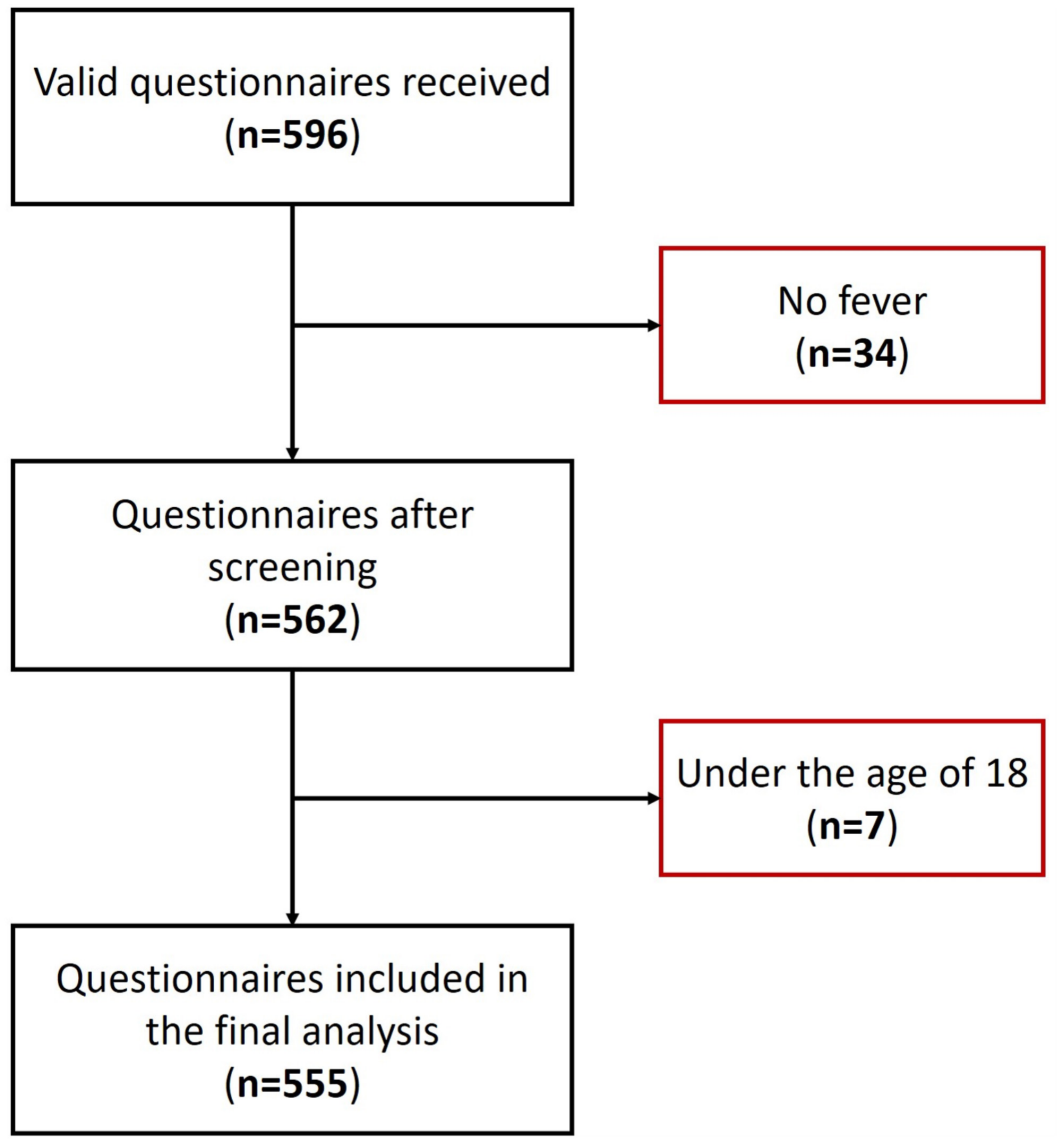
Flow diagram of questionnaires screening.

A total of 596 questionnaires were collected for this study, with 41 questionnaires excluded due to the absence of fever or the participants being under 18 years of age (Figure 1). Consequently, 555 valid questionnaires were included for analysis. The demographic characteristics of the participants are detailed in Table 1. The majority of participants were female, accounting for 71.7% (398 cases), with 55.5% (308 cases) falling within the 30–49 age bracket. The average age of participants was 39.8 ± 12.7 years. Moreover, the peak fever temperature recorded was 38.7 ± 0.7 °C, and the mean fever duration was 2.5 ± 1.6 days. A substantial proportion of participants, constituting 67.2% (373 cases), did not present with any comorbidities. Geographically, the participants were widely distributed across China, representing 25 provinces (Supplementary Table S1). Beijing emerged with the highest participant count, encompassing 68.1% (378 cases) of the total cohort.

**Table 1:** Basic demographic characteristics of participants.

**Characteristic**	**Total (*N* = 555)**	**Gender**		***P* value**
		**Male (*****N*** = **157)**	**Female (*****N*** = **398)**	
**Age**				
Total (yr), Median (Q1, Q3)	38 (30.5, 48)	36 (28, 46)	39 (32, 50)	< 0.001^a^
18–29, *n* (%)	121 (21.8)	46 (29.3)	75 (18.8)	< 0.01^b^
30–49, *n* (%)	308 (55.5)	89 (56.7)	219 (55)	
≥50, *n* (%)	126 (22.7)	22 (14)	104 (26.1)	
**Maximum body temperature** >**39** °**C**, ***n*** **(%)**				
Yes	130 (23.4)	44 (28)	86 (21.6)	0.108^b^
No	425 (76.6)	113 (72)	312 (78.4)	
**Duration of fever** >**3 days**, ***n*** **(%)**				0.381^b^
Yes	81 (14.6)	19 (12.1)	62 (15.6)	
No	472 (85.0)	138 (87.9)	334 (83.9)	
Unclear	2 (0.4)	0 (0)	2 (0.5)	
**Co-morbidities**, ***n*** **(%)**				0.659^b^
Yes	181 (32.6)	48 (30.6)	133 (33.4)	
No	373 (67.2)	109 (69.4)	264 (66.3)	
Unclear	1 (0.2)	0 (0)	1 (0.3)	

^a^Mann–Whitney test.

^b^Chi-square test.

### 3.2 Maximum body temperature and duration of fever at different age

**Figure 2:**
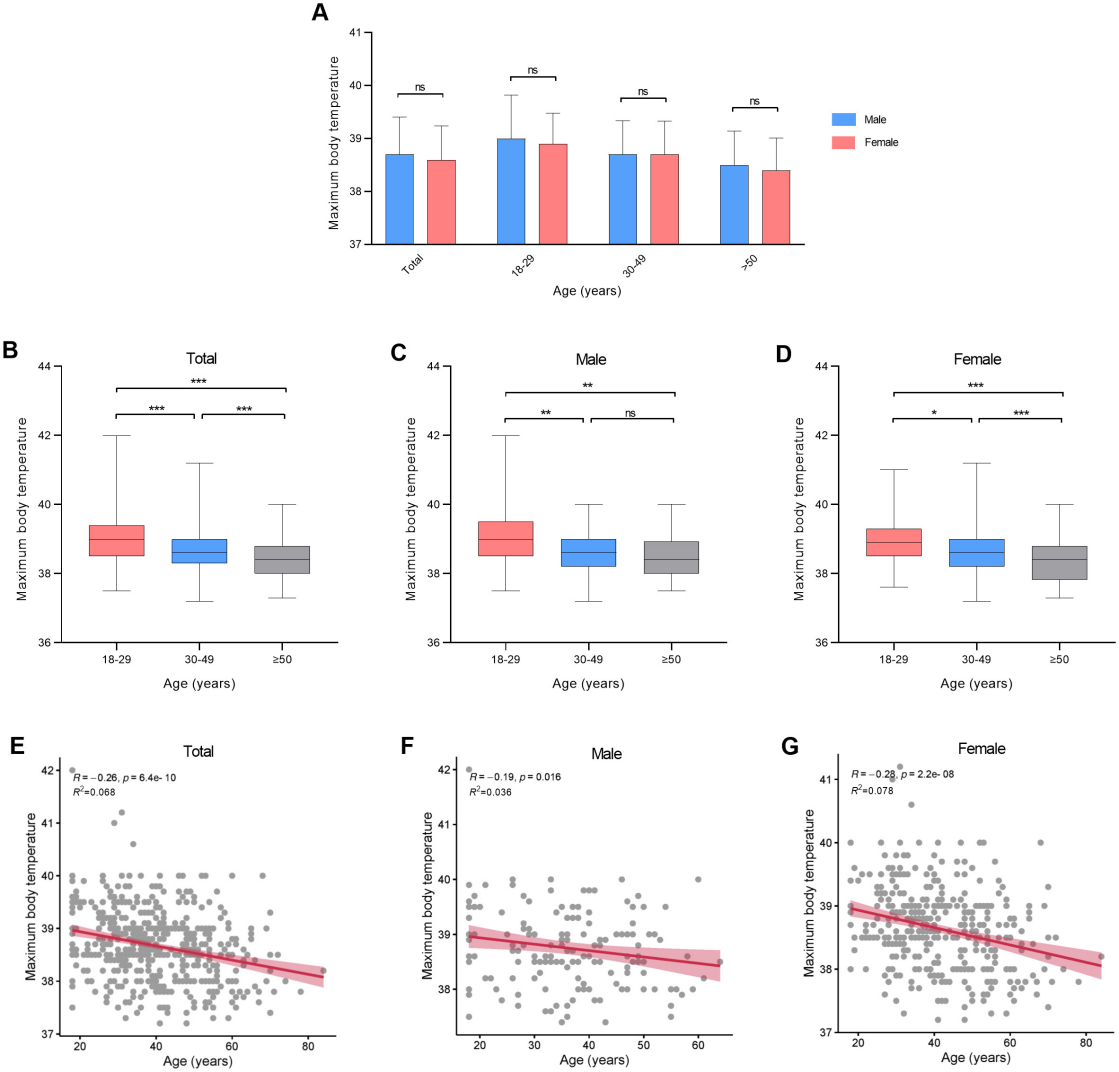
Maximum body temperature at different ages in patients infected with SARS-CoV-2. **(A)** Maximum body temperature between genders. **(B–D)** Maximum body temperature between different age groups. **(E–G)** Correlation between maximum body temperature and age. *p < 0.05, **p < 0.01, ***p < 0.001, as indicated. ns, no significant difference.

**Figure 3:**
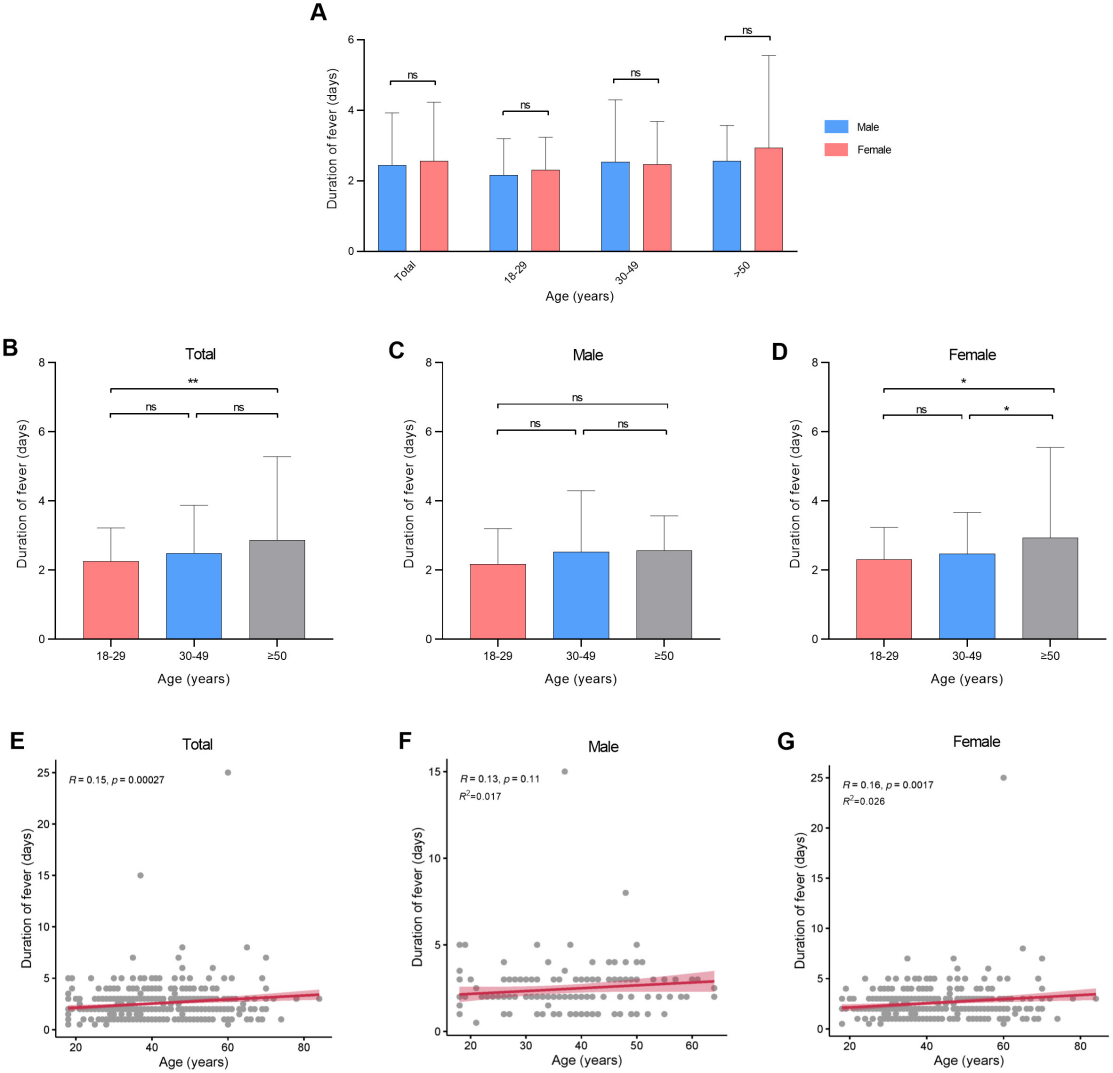
Duration of fever at different ages in patients infected with SARS-CoV-2. **(A)** Duration of fever between genders. **(B–D)** Duration of fever between different age groups. **(E–G)** Correlation between duration of fever and age. *p < 0.05, **p < 0.01, as indicated. ns, no significant difference.

There were no statistical variances in maximum body temperature observed between males and females across all age categories (Figure 2A, Table 2). Furthermore, age and sex did not exhibit a significant interaction effect (*p* = 0.711). However, notable distinctions were evident in maximum body temperature across different age groups within all genders (Figures 2B–D). Correlation analysis revealed a linear relationship between age and maximum body temperature, with negative correlations established for the overall cohort (*R* = −0.26, *p* < 0.001), males (*R* = −0.19, *p* = 0.016), and females (*R* = −0.28, *p* < 0.001; Figures 2E–G). Furthermore, no significant differences were detected in fever duration between males and females across all age brackets (Figure 3A, Table 2). Similarly, there was no statistically significant interaction between age and sex (*p* = 0.548). Individuals aged 50 years or above depicted a prolonged fever duration compared to those aged below 30 years (Figure 3B). Conversely, no significant discrepancies were observed in fever duration among different age groups within the male subgroup (Figure 3C). In contrast, females exhibited a positive association between older age and prolonged fever duration (Figure 3D). Correlation analysis pinpointed a positive correlation solely in females between age and fever duration (*R* = 0.16, *p* = 0.0017; Figures 3E–G).

**Table 2:** Maximum body temperature and duration of fever.

**Characteristic**	**Total (*N* = 555)**	**Gender**		***P* value**
		**Male (*****N*** = **157)**	**Female (*****N*** = **398)**	
**Maximum body temperature (**°**C), Median (Q1, Q3)**				
18–29 years	39 (38.5, 39.4)	39 (38.5, 39.5)	38.9 (38.5, 39.3)	0.44
30–49 years	38.6 (38.3, 39)	38.6 (38.3, 39)	38.6 (38.25, 39)	0.912
≥50 years	38.4 (38, 38.8)	38.4 (38, 38.9)	38.4 (37.9, 38.8)	0.672
Total	38.6 (38.2, 39)	38.6 (38.2, 39.2)	38.6 (38.2, 39)	0.161
**Duration of fever (days), Mean** ±**SD**				
18–29 years	2.26 ± 0.97	2.17 ± 1.03	2.31 ± 0.93	0.318
30–49 years	2.50 ± 1.38	2.54 ± 1.76	2.48 ± 1.20	0.86
≥50 years	2.88 ± 2.41	2.57 ± 1.00	2.94 ± 2.61	0.838
Total	2.53 ± 1.61	2.44 ± 1.49	2.57 ± 1.66	0.308

### 3.3 Comorbidities among participants

**Figure 4:**
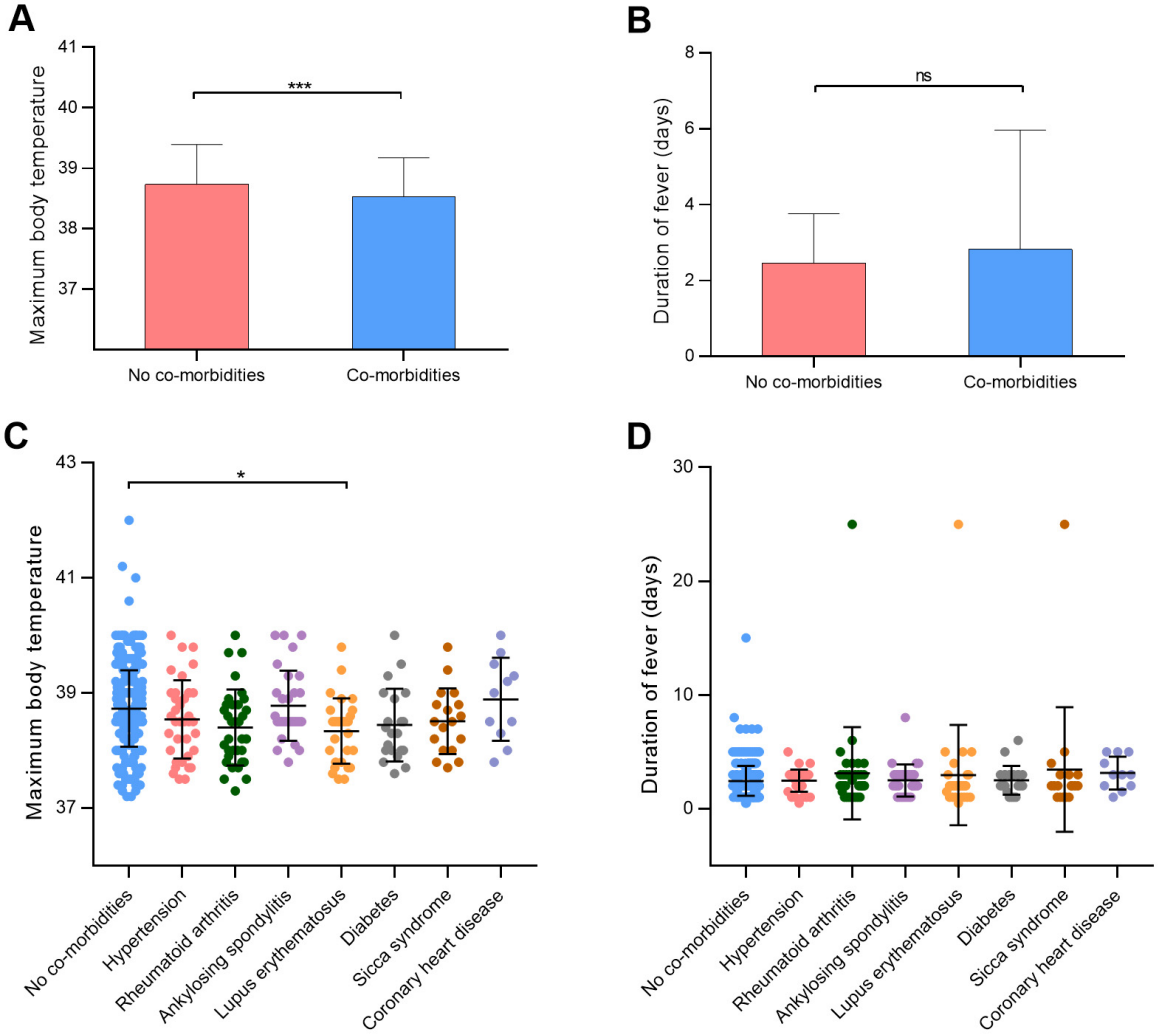
Effects of comorbidities on maximum body temperature in patients infected with SARS-CoV-2. **(A, B)** Maximum body temperature and duration of fever between participants with or without comorbidities. **(C, D)** Maximum body temperature and duration of fever in different comorbidity groups. *p < 0.05, ***p < 0.001, as indicated. ns, no significant difference.

Table 3 illustrates the distribution of comorbidities among participants in the study. Among the cohort, 182 participants (32.8%) presented with comorbidities. The top five comorbidities identified were hypertension (6.7%, 37 cases), rheumatoid arthritis (6.1%, 34 cases), ankylosing spondylitis (5.4%, 30 cases), lupus erythematosus (5.2%, 29 cases), and diabetes (3.8%, 21 cases). Ankylosing spondylitis emerged as the most prevalent comorbidity among males (13.4% in males, 2.3% in females), while rheumatoid arthritis was predominantly noted in females (2.6% in males, 7.5% in females). Significant disparities were observed in maximum body temperature between participants with and without comorbidities (*p* < 0.001; Figure 4A). Conversely, the presence of comorbidities did not impact the duration of fever (*p* = 0.74; Figure 4B). Notably, individuals with lupus erythematosus displayed a notably lower maximum body temperature compared to those without comorbidities (*p* = 0.04; Figure 4C). Furthermore, no significant distinctions were detected in fever duration between participants with no comorbidities and those with comorbidities (Figure 4D).

**Table 3:** Co-morbidities among participants.

**Disease, *n* (%)**	**Total (*N* = 555)**	**Gender**	
		**Male (*****N*** = **157)**	**Female (*****N*** = **398)**
Hypertension	37 (6.67)	13 (8.28)	24 (6.03)
Rheumatoid arthritis	34 (6.13)	4 (2.55)	30 (7.54)
Ankylosing spondylitis	30 (5.41)	21 (13.38)	9 (2.26)
Lupus erythematosus	29 (5.23)	2 (1.27)	27 (6.78)
Diabetes	21 (3.78)	7 (4.46)	14 (3.52)
Sicca syndrome	18 (3.24)	2 (1.27)	16 (4.02)
Coronary heart disease	11 (1.98)	2 (1.27)	9 (2.26)
Asthma	3 (0.54)	1 (0.64)	2 (0.5)
Hyperlipidemia	2 (0.36)	2 (1.27)	0 (0)
Gout	2 (0.36)	1 (0.64)	1 (0.25)
Anaphylactic rhinitis	2 (0.36)	0 (0)	2 (0.5)
Thrombophilia	2 (0.36)	0 (0)	2 (0.5)
No co-morbidities	373 (67.21)	109 (69.43)	264 (66.33)

### 3.4 Symptoms during and after fever in participants

**Figure 5:**
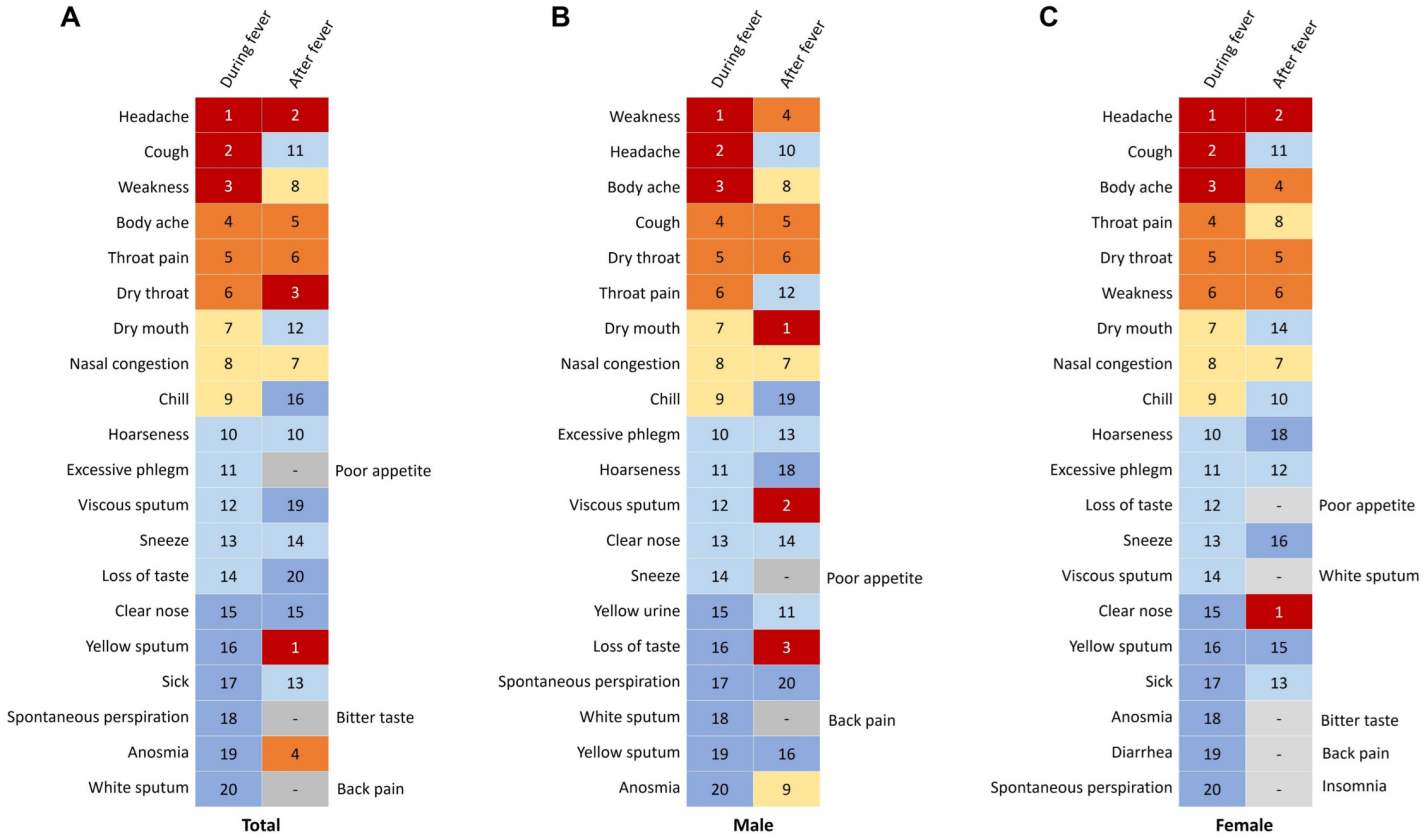
Changes in symptoms during and after fever in patients infected with SARS-CoV-2. **(A)** Symptoms in total participants. **(B)** Symptoms in males. **(C)** Symptoms in females.

Supplementary Table S2 presents the symptoms experienced during and after fever episodes among the participants. The top five symptoms observed during fever included headache (64.3%, 357 cases), cough (63.8%, 354 cases), weakness (62.2%, 345 cases), body ache (61.8%, 343 cases), and throat pain (53.5%, 297 cases) (Figure 5A). Conversely, the top five symptoms reported after fever subsided comprised cough (76.8%, 426 cases), excessive phlegm (42%, 233 cases), nasal congestion (37%, 205 cases), throat pain (36.6%, 203 cases), and dry throat (35.9%, 199 cases). Among male participants, the primary symptoms during fever included weakness, headache, and body ache, while the predominant symptoms after fever were cough, excessive phlegm, and nasal congestion (Figure 5B). In contrast, female participants predominantly reported headache, cough, and body ache as the main symptoms during fever, and cough, excessive phlegm, and throat pain as the primary symptoms after fever resolution (Figure 5C). Furthermore, during the fever phase, males exhibited higher rates of weakness than females (*p* < 0.001) but lower rates of hoarseness, taste loss, and vomiting (*p* < 0.05; Supplementary Table S2). Post-fever, males showed reduced weakness (*p* = 0.034) and significantly lower probabilities of hoarseness, appetite loss, anosmia, taste loss, nausea, and vomiting compared to females (*p* < 0.05).

## 4 Discussion

The COVID-19 pandemic has resulted in many individuals becoming victims of SARS-CoV-2 infection (15). In the course of the pandemic, swiftly understanding the common symptom patterns in the population will provide valuable insights for formulating medication and treatment plans for the uninfected population. Additionally, it can alleviate public anxiety, enabling individuals to prepare for and cope with incoming viral infections. During the COVID-19 outbreak that swept through Beijing in December 2022 (16), we promptly conducted this study to investigate symptoms during and after the fever period, aiming to enhance our understanding of the fever patterns and related symptom trends of COVID-19.

Surveillance of body temperature stands as a pivotal measure in the prompt identification of COVID-19 infection, especially among individuals who have had close contact with confirmed cases (17). Fever, a hallmark symptom in COVID-19 patients, emerges as a physiological response orchestrated by the body against viral intrusion (18). Notably, this study opted not to specify the method employed for temperature assessment, attributing this decision to the comparable efficacy of prevalent home-based temperature measurement techniques, such as axillary mercury thermometers and infrared forehead thermometers, which have demonstrated overlapping measurement outcomes and are deemed reliable (19). The survey targeted individuals aged between 18 and 70 for various reasons. Individuals below 18 years of age are still in the process of physical development, which makes their bodies more unique, and there is a possibility that they may not provide clear responses that could affect the accuracy of the results. Conversely, the elderly population aged over 70 may exhibit reduced smartphone usage rates or encounter declines in visual or cognitive capacities, potentially introducing inaccuracies in their reported data.

This study uncovered that the average duration of fever among adult patients afflicted with SARS-CoV-2 amounted to ~2.5 days, with an average peak body temperature of 38.7 °C among fever-stricken individuals. These findings markedly deviate from those reported in extant literature (20, 21), potentially attributed to the study's selective inclusion criteria specifying the presence of fever in COVID-19 patients, with a stipulated threshold for inclusion set at a minimum body temperature of ≥4 °C. In contrast, previous studies encompassed a broader patient cohort that included individuals without fever symptoms. For example, Yang et al.'s (22) study showed that the fever duration of COVID-19 patients in Wuhan, China, was 4.2 days, with the highest body temperature reaching 37.6 °C, which differs from our figures. This may be because the study was conducted at the beginning of 2020 when COVID-19 had just emerged, and the strains and characteristics of SARS-CoV-2 infections at that time were already significantly different from those in 2022. Moreover, given the purportedly milder symptomatology associated with the Omicron variant, numerous patients opt for self-care at home upon symptom onset (23). The survey cohort predominantly comprised such self-managing individuals, further distinguishing the data collected in this study from prior reports focusing on the Delta variant and hospitalized patients.

The observed negative correlation between age and peak body temperature could be attributed to several age-related physiological changes. Immune senescence, characterized by a diminished immune response in the elderly, might lead to a blunted pyrogenic reaction to SARS-CoV-2 infection (24). Moreover, the thermoregulatory center of the hypothalamus deteriorates with age, leading to the need for higher concentrations of pyrogens for a response, making fever less likely to occur (25). Additionally, the basal body temperature of older adults is inherently lower than that of younger individuals, which may also objectively contribute to the impression of “difficulty in developing fever” (26). The positive association between age and fever duration specifically in female participants presents an intriguing finding. This might be linked to sex-specific differences in immune and inflammatory responses that evolve with age (27). The hormonal changes associated with menopause, such as the decline in estrogen which has known immunomodulatory effects, could potentially alter the kinetics of the febrile response and prolong the resolution of inflammation (28). While this specific finding requires further validation, it highlights the importance of considering sex and age as interactive variables in infectious disease research. Individuals with lupus erythematosus show a tendency toward lower maximum body temperatures after being infected with SARS-CoV-2. However, this finding is based on a small subgroup and should be interpreted with caution as a preliminary observation requiring validation in larger, specifically designed studies.

Our study identifies fever, myalgia, cough, fatigue, and headache as cardinal symptoms manifested during the early disease stages of COVID-19. Notably, post-fever resolution, respiratory symptoms including cough, productive sputum, nasal congestion, and pharyngodynia, alongside persistent fatigue, emerge as dominant clinical features. These findings suggest that antipyretic-analgesic agents should be prioritized during the early febrile phase of infectious diseases to alleviate symptoms, while following defervescence, attention should shift to the judicious use of cough suppressants, expectorants, and nasal decongestants (29). The description of symptoms during and after the febrile phase provides valuable cross-sectional information on symptom prevalence at these distinct time points. However, as this is cross-sectional data collected via self-report, it cannot strictly infer the temporal sequence of symptom evolution at the individual level. Given the study's exclusive focus on fever-presenting COVID-19 patients, divergences may be observed in the primary symptomatology at disease onset compared to other research studies. Furthermore, varying regional ecological conditions can contribute to the diverse symptom profiles seen in COVID-19 cases.

This study possesses several notable strengths. It represents a focused exploration of fever characteristics within the Chinese population affected by COVID-19, a domain with relatively limited prior research. This targeted investigation augments our comprehension of the symptomatology exhibited by individuals afflicted with SARS-CoV-2 in this demographic, offering valuable insights for the implementation of potential control strategies during future outbreaks. The study underscores the influence of age and gender on fever presentations among the COVID-19 cohort and highlights the impact of specific comorbidities on fever intensity. Furthermore, an examination of symptom dynamics pre-and post-fever onset in SARS-CoV-2 infection has implications for healthcare resource allocation in epidemic settings.

It is essential to consider the predominant COVID-19 strain when interpreting the outcomes of this study. Regrettably, precise testing of the study participants for viral strains was unfeasible at the time, presenting a significant challenge in data acquisition. News reports circulating during the study period suggested a heightened probability of the prevalence of the Omicron variant in China. The rapid dissemination of the Omicron variant, distinguished by heightened transmissibility compared to earlier strains, precipitated a swift outbreak within China (30, 31). The prevailing epidemic circumstances impeded the identification of viral subtypes among the patients, contributing to a limitation of this study.

The recruitment of participants primarily through the WeChat platform, a smartphone-based application, inevitably introduced selection bias into our study cohort. This is reflected in the overrepresentation of females (71.7%), individuals aged 30–49 years (55.5%), and residents of Beijing (68.1%). Consequently, our findings may not be fully generalizable to the broader Chinese population, particularly to elderly individuals, males, residents of rural areas, or those with limited access to or proficiency with digital technology. This bias potentially limits the external validity of our study. Furthermore, this specific demographic composition raises the possibility that the observed associations, such as the negative correlation between age and peak body temperature and the positive correlation between age and fever duration in females, might be influenced by the sampling framework. For instance, the underrepresentation of older age groups, especially those over 70 who may exhibit different fever responses and have lower smartphone usage rates, could lead to an underestimation of the true effect of advanced age on fever characteristics. Similarly, the predominance of a specific demographic (urban, middle-aged, tech-savvy) might affect the magnitude and even the direction of the associations we reported. Therefore, the results of this study should be interpreted as primarily applicable to a relatively young, urban-dwelling, female-predominant population with proficiency in using smartphones. Caution is advised when extrapolating these findings to other demographic groups.

A further major limitation of this study pertains to potential measurement and information biases. Firstly, the core outcome variables—fever duration and peak body temperature—were based on self-reported data without objective verification. This reliance on participant recall is susceptible to inaccuracies; recall bias may be particularly pronounced for the duration of fever, potentially leading to misclassification. Secondly, while we acknowledged the use of common household devices (axillary or infrared thermometers), the lack of a standardized and validated method for measuring body temperature likely introduced non-differential misclassification and measurement error. The variation between axillary and forehead temperature measurements, and even between different device brands, could have added uncontrolled variability to the peak temperature data. Thirdly, the identification of comorbidities and symptoms was also based on self-reporting without clinical validation. This method is vulnerable to both under-reporting and over-reporting, leading to potential misclassification of these variables. These measurement errors represent significant weaknesses as they can attenuate the observed effect sizes, potentially causing true associations to bias toward the null and making it more difficult to detect significant relationships. For instance, the random error in self-reported temperature measurement could dilute the estimated correlation between age and peak temperature. Conversely, in some cases, non-random error could theoretically create spurious associations. While we cannot definitively quantify the impact, these biases undoubtedly affect the precision and potentially the accuracy of our findings, and they must be considered when interpreting the results.

Importantly, our analysis did not adjust for several key potential confounding variables, which considerably limits the internal validity of the observed associations. Firstly, we did not collect data on vaccination status including number of doses, vaccine type, and timing of last vaccination, which is a well-established factor influencing COVID-19 symptom severity and presentation. Secondly, while the study was conducted during the Omicron-dominant period in Beijing, we did not perform laboratory confirmation of the infecting viral variant for each participant, leaving the possibility of co-circulation of other variants unaccounted for. Thirdly, data on the use of symptomatic treatments, such as antipyretics and cold medicines, which directly impact fever duration and peak temperature, were not collected. Fourthly, participants' prior SARS-CoV-2 infection history was not ascertained. Previous infections could alter the immune response and symptom profile of a subsequent infection. Finally, the known age-related decline in basal metabolic rate and baseline body temperature in the elderly could be a biological confounder for the observed negative correlation between age and peak temperature, suggesting the association might not be solely attributable to the immune response to SARS-CoV-2. The inability to measure and control for these factors is a critical weakness. Their omission means we cannot rule out the possibility that the observed associations are partly or wholly explained by these confounders. For example, the negative correlation between age and peak temperature might be influenced by a higher vaccination rate or more frequent antipyretic use in older age groups, or by their physiologically lower baseline temperature. Conversely, the lack of adjustment could also lead to residual confounding, potentially resulting in spurious associations. Therefore, the relationships reported in our study should be interpreted as unadjusted associations rather than causal relationships, and our findings necessitate verification in studies designed to collect and adjust for these critical confounding variables.

This study is subject to several other noteworthy limitations. Firstly, owing to the exigency of swiftly accruing pertinent data during a unique timeframe, the survey questionnaire was intentionally streamlined, resulting in limited data acquisition. Secondly, the study exclusively confirmed COVID-19 diagnosis without further probing for influenza or common cold co-infections, implying a potential overlap in symptoms from these illnesses. Furthermore, the study did not investigate the medication use after SARS-CoV-2 infection as well as the vaccination status of COVID-19 vaccines, leaving room for unexplored confounding factors that may influence the research outcomes.

## 5 Conclusion

In conclusion, our study highlights the significant impact of age on both the maximum body temperature and duration of fever in COVID-19 patients. Notably, individuals with lupus erythematosus demonstrate a trend toward lower maximum body temperatures following SARS-CoV-2 infection. Noteworthy differences in symptomatology are observed between the fever phase and post-fever period in COVID-19 patients. These insights help strengthen our understanding of the symptoms of SARS-CoV-2 infection, providing valuable lessons for potential similar viral infections in the future. It is important to acknowledge the potential influence of various confounding factors on our findings, which may limit their generalizability. Therefore, we advise exercising caution in interpreting the conclusions drawn from this study.

## Data Availability

The original contributions presented in the study are included in the article/Supplementary material, further inquiries can be directed to the corresponding authors.
