# Supplementary material for: Fever duration, peak temperature and associated symptoms in Chinese adults with SARS-CoV-2 infection: a cross-sectional study
Source: Front Med (Lausanne). 2025 Nov 6;12:1665460. doi: 10.3389/fmed.2025.1665460 (PMC12631222; doi:10.3389/fmed.2025.1665460)
Supplement: Supplementary file 1 [file Data_Sheet_1.pdf]

Table S1. The geographical distribution of participants.

Characteristic	Total (N=555)	Gender	
		Male (N=157)	Female (N=398)
**Location, n (%)**			
Anhui	2 (0.36)	1 (0.64)	1 (0.25)
Beijing	378 (68.11)	90 (57.32)	288 (72.36)
Chongqing	4 (0.72)	3 (1.91)	1 (0.25)
Guangdong	1 (0.18)	1 (0.64)	0 (0)
Guangxi	1 (0.18)	0 (0)	1 (0.25)
Guizhou	1 (0.18)	0 (0)	1 (0.25)
Hebei	48 (8.65)	14 (8.92)	34 (8.54)
Henan	12 (2.16)	4 (2.55)	8 (2.01)
Heilongjiang	3 (0.54)	2 (1.27)	1 (0.25)
Hunan	2 (0.36)	1 (0.64)	1 (0.25)
Inner mongolia	2 (0.36)	1 (0.64)	1 (0.25)
Jilin	1 (0.18)	1 (0.64)	0 (0)
Jiangsu	6 (1.08)	0 (0)	6 (1.51)
Jiangxi	3 (0.54)	1 (0.64)	2 (0.5)
Liaoning	10 (1.80)	4 (2.55)	6 (1.51)
Ningxia	1 (0.18)	0 (0)	1 (0.25)
Shandong	45 (8.11)	15 (9.55)	30 (7.54)
Shanxi	6 (1.08)	5 (3.18)	1 (0.25)
Shaanxi	4 (0.72)	2 (1.27)	2 (0.5)
Shanghai	2 (0.36)	1 (0.64)	1 (0.25)
Sichuan	3 (0.54)	2 (1.27)	1 (0.25)
Tianjin	9 (1.62)	6 (3.82)	3 (0.75)
Xizang	1 (0.18)	0 (0)	1 (0.25)
Xinjiang	2 (0.36)	1 (0.64)	1 (0.25)
Zhejiang	3 (0.54)	1 (0.64)	2 (0.5)

Abroad	4 (0.72)	1 (0.64)	3 (0.75)

Table S2. Symptoms during and after fever in participants.

Disease, n (%)	Total (N=555)	Gender		*P* value
		Male (N=157)	Female (N=398)	
**During fever, n (%)**				
Headache	357 (64.32)	95 (60.51)	262 (65.83)	0.239
Cough	354 (63.78)	94 (59.87)	260 (65.33)	0.229
Weakness	345 (62.16)	135 (85.99)	210 (52.76)	**<0.001**
Body ache	343 (61.80)	95 (60.51)	248 (62.31)	0.694
Throat pain	297 (53.51)	77 (49.04)	220 (55.28)	0.185
Dry throat	294 (52.97)	83 (52.87)	211 (53.02)	0.975
Dry mouth	240 (43.24)	71 (45.22)	169 (42.46)	0.554
Nasal congestion	236 (42.52)	68 (43.31)	168 (42.21)	0.813
Chill	229 (41.26)	66 (42.04)	163 (40.95)	0.815
Hoarseness	206 (37.12)	44 (28.03)	162 (40.70)	**0.005**
Excessive phlegm	178 (32.07)	55 (35.03)	123 (30.90)	0.348
Viscous sputum	143 (25.77)	41 (26.11)	102 (25.63)	0.906
Sneeze	137 (24.68)	32 (20.38)	105 (26.38)	0.14
Loss of taste	135 (24.32)	28 (17.83)	107 (26.88)	**0.025**
Clear nose	134 (24.14)	39 (24.84)	95 (23.87)	0.81
Yellow sputum	116 (20.90)	27 (17.20)	89 (22.36)	0.178
Sick	106 (19.10)	22 (14.01)	84 (21.11)	0.056
Spontaneous perspiration	105 (18.92)	28 (17.83)	77 (19.35)	0.682
Anosmia	104 (18.74)	25 (15.92)	79 (19.85)	0.286
White sputum	102 (18.38)	28 (17.83)	74 (18.59)	0.835
Diarrhea	101 (18.20)	22 (14.01)	79 (19.85)	0.109
Yellow urine	80 (14.41)	29 (18.47)	51 (12.81)	0.087
Aversion to wind	71 (12.79)	17 (10.83)	54 (13.57)	0.384
Emesis	64 (11.53)	10 (6.37)	54 (13.57)	**0.017**
Hypochondriac pain	55 (9.91)	12 (7.64)	43 (10.80)	0.262

Constipation	38 (6.85)	8 (5.10)	30 (7.54)	0.305
Turbid nose	38 (6.85)	11 (7.01)	27 (6.78)	0.926
Abdominal distension	35 (6.31)	7 (4.46)	28 (7.04)	0.261
**After fever, n (%)**				
Cough	426(76.76)	113(71.97)	313(78.64)	0.094
Excessive phlegm	233(41.98)	70(44.59)	163(40.95)	0.435
Nasal congestion	205(36.94)	57(36.31)	148(37.19)	0.847
Throat pain	203(36.58)	50(31.85)	153(38.44)	0.146
Dry throat	199(35.86)	52(33.12)	147(36.93)	0.399
Weakness	186(33.51)	42(26.75)	144(36.18)	**0.034**
Viscous sputum	174(31.35)	49(31.21)	125(31.41)	0.964
Dry mouth	162(29.19)	38(24.2)	124(31.16)	0.105
Yellow sputum	130(23.42)	36(22.93)	94(23.62)	0.863
Hoarseness	125(22.52)	23(14.65)	102(25.63)	**0.005**
Poor appetite	123(22.16)	24(15.29)	99(24.87)	**0.014**
Anosmia	122(21.98)	22(14.01)	100(25.13)	**0.004**
Loss of taste	119(21.44)	19(12.1)	100(25.13)	**<0.001**
White sputum	118(21.26)	30(19.11)	88(22.11)	0.436
Clear nose	115(20.72)	35(22.29)	80(20.1)	0.566
Headache	112(20.18)	26(16.56)	86(21.61)	0.182
Sneeze	99(17.84)	26(16.56)	73(18.34)	0.622
Bitter taste	74(13.33)	14(8.92)	60(15.08)	0.055
Body ache	69(12.43)	23(14.65)	46(11.56)	0.32
Back pain	69(12.43)	21(13.38)	48(12.06)	0.672
Chill	63(11.35)	16(10.19)	47(11.81)	0.588
Insomnia	63(11.35)	15(9.55)	48(12.06)	0.402
Diarrhea	46(8.29)	10(6.37)	36(9.05)	0.303
Sick	44(7.93)	4(2.55)	40(10.05)	**0.003**
Spontaneous perspiration	38(6.85)	9(5.73)	29(7.29)	0.514

Aversion to wind	32(5.77)	9(5.73)	23(5.78)	0.983
Yellow urine	31(5.59)	13(8.28)	18(4.52)	0.083
Turbid nose	30(5.41)	5(3.18)	25(6.28)	0.146
Emesis	21(3.78)	0(0)	21(5.28)	**0.003**
Abdominal distension	19(3.42)	6(3.82)	13(3.27)	0.746
Constipation	19(3.42)	6(3.82)	13(3.27)	0.746
Hypochondriac pain	12(2.16)	3(1.91)	9(2.26)	0.798
